# Polymeric Nanocapsules Loaded with Lidocaine: A Promising Formulation for Topical Dental Anesthesia

**DOI:** 10.3390/ph17040485

**Published:** 2024-04-10

**Authors:** Camila Batista da Silva, Cleiton Pita dos Santos, Luciano Serpe, Jonny Burga Sanchez, Luiz Eduardo Nunes Ferreira, Nathalie Ferreira Silva de Melo, Francisco Carlos Groppo, Leonardo Fernandes Fraceto, Maria Cristina Volpato, Michelle Franz-Montan

**Affiliations:** 1 Department of Biosciences, Piracicaba Dental School, Universidade Estadual de Campinas, Av. Limeira, 901, Piracicaba, SP 13414-903, Brazil; camilaca@unicamp.br (C.B.d.S.); cleitonpita@yahoo.com.br (C.P.d.S.); lucianoserpe@yahoo.com.br (L.S.); jonnyburga@gmail.com (J.B.S.); luiz.enferreira@gmail.com (L.E.N.F.); fcgroppo@unicamp.br (F.C.G.); mcristinavolpato@gmail.com (M.C.V.); 2 Laboratory of Inflammation and Immunology, Guarulhos University, Guarulhos, SP 07023-070, Brazil; 3 Department of Environmental Engineering, São Paulo State University, Sorocaba, SP 18087-180, Brazil; nathaliemelo@gmail.com (N.F.S.d.M.); leonardo.fraceto@unesp.br (L.F.F.)

**Keywords:** dentistry, anesthetic efficacy, topical anesthesia, lidocaine, poly(epsilon-caprolactone) nanocapsules, cytotoxicity, permeation

## Abstract

Lidocaine is the most commonly used local anesthetic worldwide, known for its rapid onset and moderate duration of anesthesia. However, it is short-lived and does not effectively promote effective topical anesthesia in the oral cavity when used alone. Our aim was to investigate whether an approximate 50% encapsulation of lidocaine in poly(ε-caprolactone) nanocapsules (LDC-Nano) would be able to increase its permeation and analgesic efficacy and reduce cytotoxicity. In this study, we characterized LDC-Nano and conducted MTT tests with HaCaT cells to assess their in vitro cytotoxicity. Additionally, in vitro permeation assays across the pig esophageal epithelium and the anesthetic efficacy of the hind paw incision model in rats were performed. Plain lidocaine (LDC) was compared with LDC-Nano and lidocaine hydrochloride plus epinephrine (LDC-Epi). The physicochemical characteristics of LDC-Nano were satisfactory (pH: 8.1 ± 0.21; polydispersity index: 0.08 ± 0.01; mean diameter (nm): 557.8 ± 22.7; and encapsulation efficiency (%): 51.8 ± 1.87) and remained stable for up to 4 months. LDC-Nano presented similar in vitro cytotoxicity to LDC but was higher than LDC-Epi (LD_50_: LDC = 0.48%; LDC-Nano = 0.47%; and LDC-Epi = 0.58%; *p* < 0.0001). Encapsulation increased the permeability coefficient about 6.6 times and about 7.5 the steady-state flux of lidocaine across the mucosal epithelium. Both encapsulation and epinephrine improved anesthesia duration, with epinephrine demonstrating superior efficacy (100% of animals were anesthetized up to 100, 30, and 20 min when LDC-Epi, LDC-nano, and LDC were used, respectively). Although LDC-Epi demonstrated superior in vivo anesthetic efficacy, the in vitro permeation and cytotoxicity of LDC-Nano indicate promising avenues for future research, particularly in exploring its potential application as a topical anesthetic in the oral cavity.

## 1. Introduction

Local anesthetics play a crucial role in pain control during various medical and dental procedures. Lidocaine, an amide local anesthetic, is recognized as a gold standard in local anesthesia and is the most common and extensively utilized [1]. Although it presents a rapid onset and effective anesthetic properties, it has certain limitations, especially in dentistry [2]. For example, the duration of pulpal anesthesia is restricted to 10 min when administered as a plain solution, necessitating the inclusion of a vasoconstrictor to perform dental treatments [2]. While lidocaine serves well as a topical anesthetic for painless punctures, it does not allow a pain-free injection experience during local anesthesia, particularly in sensitive areas such as the palate [3].

Drug delivery systems have been investigated to reduce toxicity and improve the bioavailability and clinical efficacy of drugs, including local anesthetics [4,5]. The group led by Prof. Leonardo Fraceto has been focusing on developing polymeric nanoparticles for the delivery of local anesthetics with enhanced efficacy and reduced cytotoxicity [6,7,8,9,10]. Nanocapsules present a lipophilic or hydrophilic nucleus surrounded by a polymeric matrix with a high drug encapsulation efficiency, as drugs can be encapsulated in the nucleus, embedded in the polymeric capsule, or adsorbed to the surface [11,12].

A previous study with lidocaine loaded in poly(epsilon-caprolactone) nanospheres showed an encapsulation efficiency of 93% with good physical properties and potential application in controlled release for pain management [8]. Nevertheless, a higher encapsulation efficiency may result in a moderate or prolonged onset of action, as evidenced by lidocaine in solid lipid nanoparticles [13].

To address this issue, our group recently proposed a reduction in the encapsulation of articaine, which resulted in a fast onset of analgesia evaluated through a hypernociceptive model in rats [10]. In this context, we hypothesized that a reduction of approximately 50% in the encapsulation efficiency of lidocaine could lead to a similar effect. Concurrently, this adjustment retains the advantageous aspects of drug encapsulation, such as increased tissue permeability, thereby extending the duration of anesthesia while mitigating potential toxicity [14]. Thus, we aimed to develop a formulation with approximately 50% encapsulation of lidocaine in poly(ε-caprolactone) nanocapsules (LDC-Nano) and evaluate its permeation capacity, analgesic efficacy, and cytotoxicity. 

## 2. Results

The results from this study were previously reported in a PhD thesis [14].

### 2.1. Nanoparticle Characterization

#### 2.1.1. Particle Size, Encapsulation Efficiency, and Stability

**Figure 1:**
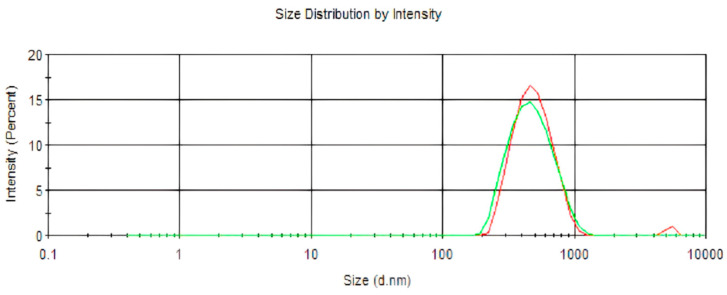
LDC-Nano is represented in red, while Nano is shown in green in the size distribution obtained through the PCS technique.

**Figure 2:**
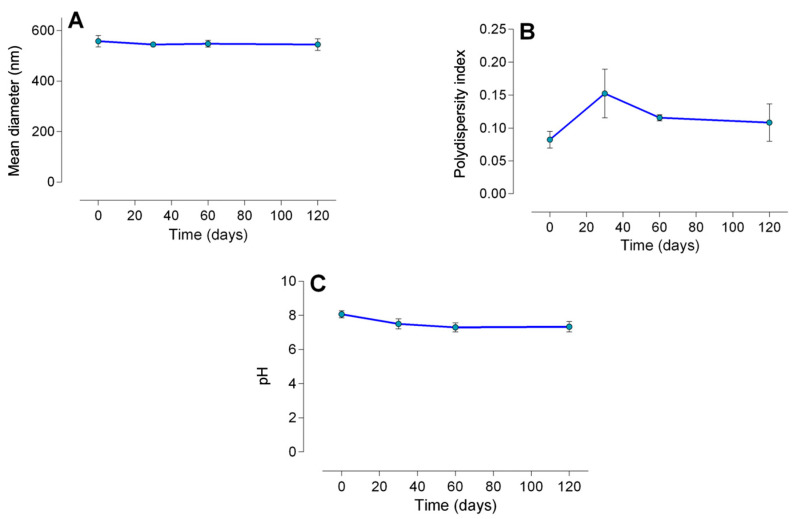
Mean diameter in nm (**A**), polydispersity index (**B**), and pH (**C**) of the LDC-Nano evaluated over 4 months of storage at room temperature.

The size of the nanoparticles remained unaltered upon the encapsulation of lidocaine in poly(epsilon-caprolactone) nanocapsules (*p* = 0.13) (Table 1; Figure 1). However, the polydispersity index showed a reduction (*p* = 0.008), and the pH exhibited an increase (*p* < 0.0001), upon the encapsulation of lidocaine. As presented in Figure 2, LDC-Nano presented a notable stability with no differences in the mean diameter (*p* < 0.05) (Figure 2A), polydispersity index (*p* < 0.05) (Figure 2B), and pH (*p* < 0.05) (Figure 2C) over a period of 120 days.

#### 2.1.2. Differential Scanning Calorimetry (DSC)

**Figure 3:**
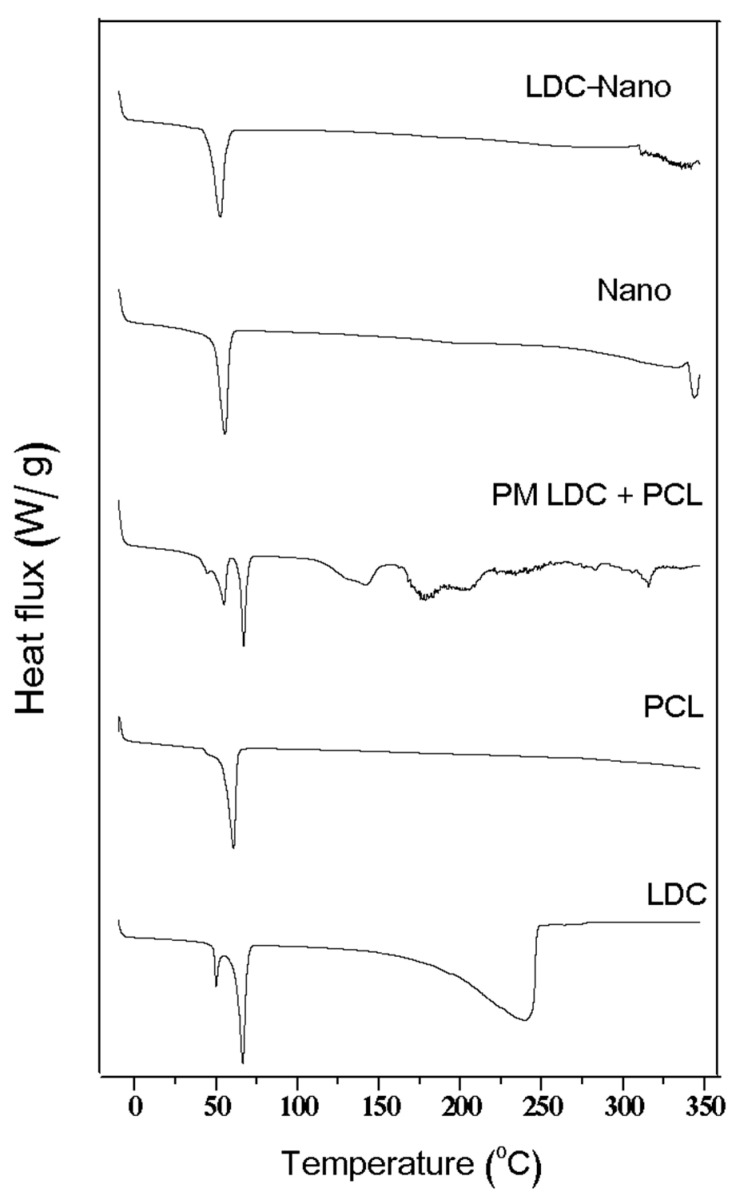
Differential scanning calorimetry (DSC) thermograms of the tested formulations and controls.

Thermograms of lidocaine encapsulated in poly(epsilon-caprolactone) nanocapsules (LDC-Nano), poly(epsilon-caprolactone) nanocapsules (Nano), a physical mixture of lidocaine and poly(epsilon-caprolactone) polymer (PCL) (PM LDC + PCL), poly(epsilon-caprolactone) (PCL), and lidocaine (LDC) are presented in Figure 3.

### 2.2. MTT Assay

**Figure 4:**
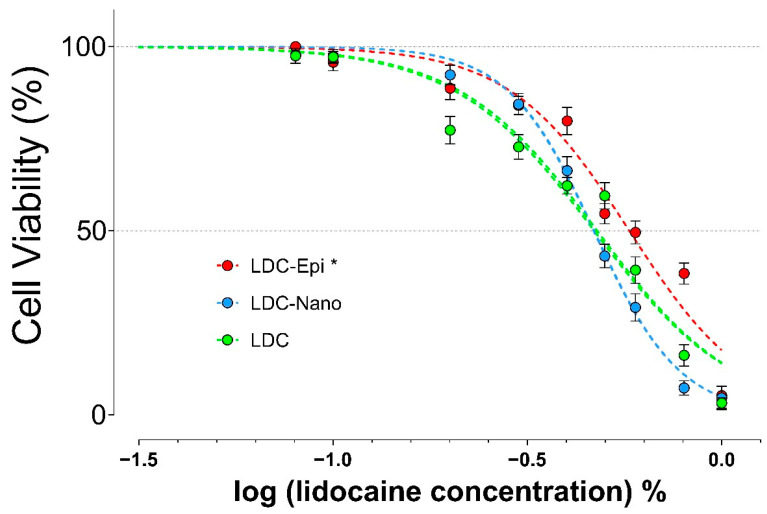
The cell viability of HaCaT cells (%, mean ± SEM) was assessed using the MTT test after contact with different concentrations of LDC, LDC-Nano, and LDC-Epi for 1 h. The non-linear fit analysis revealed statistical significance (* *p* < 0.0001 compared to LDC and LDC-Nano).

The in vitro cytotoxicity of the formulation in HaCaT cells is illustrated in Figure 4. LDC-Nano did not alter the level of lidocaine cytotoxicity (LD50: LDC = 0.48%; LDC-Nano = 0.47%; *p* = 0.54). Furthermore, the addition of epinephrine to the formulation resulted in a reduced lidocaine toxicity (LD50 LDC-Epi = 0.58%; *p* < 0.0001), indicating a potential protective effect.

### 2.3. Permeation Assay

**Figure 5:**
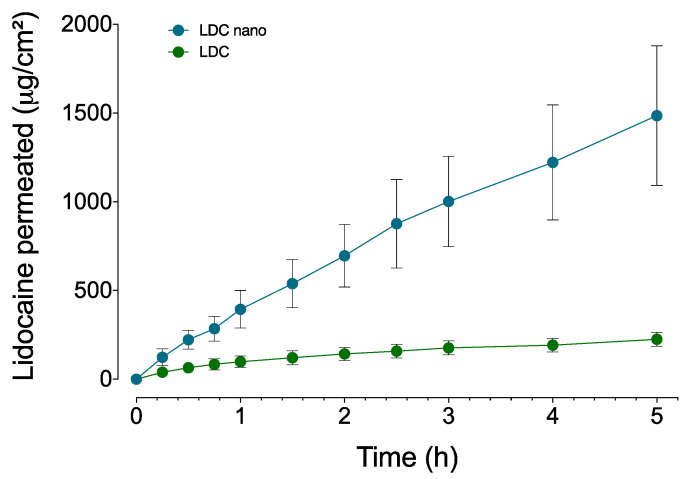
Permeation profiles of 2% LDC-Nano or 2% LDC across the pig esophageal epithelium after 300 min experiment applied under infinite dose conditions (*n* = 6; mean ± SD). Linear regression analysis between curves (*p* < 0.0001).

The permeation profiles of LDC and LDC-Nano are shown in Figure 5. The linear regression analysis showed that LDH presented a higher permeation across the buccal epithelium when associated with nanocapsules (*p* < 0.0001). Permeation parameters, as shown in Table 2, were calculated within the linear interval (between 0.25 and 5 h), with regression coefficients surpassing 0.97. The inclusion of lidocaine-loaded nanocapsules increased the lidocaine flux by 7.46 times (*p* < 0.0001) and the amount of lidocaine permeated by 6.61 times (*p* = 0.0002) (Table 2). In addition to the enhanced permeation, there was no observable lag time, indicating immediate drug transport.

### 2.4. Anesthetic Efficacy

**Figure 6:**
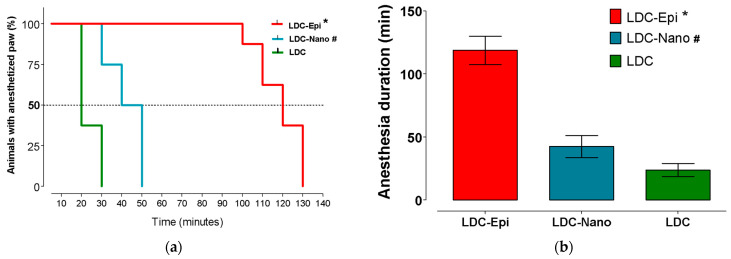
Anesthetic efficacy after the infiltration of LDC, LDC-Nano, and LDC-Epi in rat paws undergoing hyperalgesia development: (**a**) anesthesia success (log-rank Mantel–Cox test; *n* = 8; * *p* < 0.0001 in relation to the other formulations; # *p* = 0.0008 in relation to LDC); (**b**) anesthesia duration (mean and standard deviation) (ANOVA and Holm–Sidak’s multiple comparisons test; *n* = 8; * *p* < 0.0001 in relation to the other formulations; # *p* = 0.0003 in relation to LDC).

All the animals were considered hyperalgesia and were submitted for assessment of anesthetic efficacy. The control formulations (NaCl and Nano) did not induce local analgesia. Anesthesia success and duration are shown in Figure 6a,b, respectively. LDC-Epi presented higher anesthetic success and longer durations in comparison to LDC-Nano and LDC (*p* < 0.0001). Moreover, LDC-nano presented higher anesthetic success and longer duration in comparison to LDC (*p* < 0.0003). 

## 3. Discussion

Polymeric systems have been developed for lidocaine delivery, where high encapsulation efficiencies (higher than 90%) in nanocapsules [15] and nanospheres [8] were observed. These systems offer advantages by improving the drug’s properties, such as permeation and absorption, and enhancing its efficacy and safety. However, in dental practice, a shorter onset of action is also desired. In this way, the present study has successfully developed a strategic polymeric nanoparticle system aimed at topical dental anesthesia. The preparation of the nanoparticles employed enabled an encapsulation of lidocaine of almost 52%, leading to a faster onset of action, increased tissue permeability, and reduced cytotoxicity against a human keratinocyte cell line.

Physical parameters, such as measurements of average diameter, polydispersity index (PDI), pH, and encapsulation efficiency, are important for obtaining information concerning the nature of colloidal systems [11]. Substantial alterations in these parameters signal the formation of aggregates and a decrease in stability [16]. The diameter of LDC-Nano was consistent with the dimensions reported in the literature for polymeric nanocapsules loaded with lidocaine [8] and articaine encapsulated in poly(epsilon-caprolactone) nanocapsules [10,17]. In fact, minor fluctuations were noted in the diameter sizes of various polymeric nanoparticles [7,8,18,19,20].

The analysis of pH also contributes to understanding nanoparticle stability. The decrease in pH could be linked to the hydrolysis of poly(epsilon-caprolactone) nanocapsules, resulting in an increase in the concentration of terminal carboxylic groups due to the relaxation of polymeric chains, as documented in previous studies [8,10,16]. In our investigation, a slight pH reduction was noted in the initial observation period, followed by stability over the subsequent two periods (60 and 120 days), consistent with previous findings [8].

Concerning the DSC thermogram analysis, the endothermic peaks for LDC and PCL are consistent with what has been reported in the literature [21]. Nano exhibited an endothermic peak, indicating that its preparation process increased the heterogeneity of the polymeric crystals, resulting in less perfect structures with a lower transition melting temperature. The absence of a melting-endothermic peak for LDC-Nano suggests that the interaction between LDC and Nano altered the crystalline network of the compound. A lidocaine peak was not observed in LDC-Nano, suggesting that lidocaine might be internally dispersed or adsorbed onto the surface of the nanocapsules [22].

The cytotoxicity of lidocaine has been investigated in the literature against different cell types, presenting an extensive result. The concentration of lidocaine leading to 50% cell lethality varies across different cell lines. Values of 0.4% and 0.09% for SH-SY5Y cells after a 10 and 20 min treatment for human neuroblastoma (SH-SY5Y) have been reported, respectively [23,24], and 3.56% for human oral mucosa fibroblasts following a 1 h exposure period [19]. Moreover, after 24 h of exposure, lidocaine at 1.6 mg/mL induced 70% cell death in adipose cells [25]. Even at clinically employed concentrations, typically 2% in infiltration solutions and up to 10% in topical formulations, lidocaine has the potential to induce tissue damage [2]. 

The duration of exposure to local anesthetics is a critical factor to consider. While dilution in tissue fluids and absorption into the bloodstream may alleviate tissue toxicity, topical applications can expose cells to the local anesthetic for an extended period of time (1 h). Our findings indicate that the toxicity of lidocaine in nanocapsules is similar to non-encapsulated local anesthetics. Moreover, the presence of epinephrine was observed to alleviate lidocaine toxicity, consistent with previous findings on articaine [10]. It is important to note that the concentrations evaluated in this in vitro study closely match the range in which lidocaine is clinically administered [2]. 

Contentious research presents contradictory findings regarding the influence of epinephrine combined with local anesthetics on cellular viability. No alteration was detected in the availability of chondrocytes when subjected to 2% lidocaine with or without epinephrine for 24 h [26]. In contrast, Braun et al. [27] illustrated augmented synoviocyte mortality following a 24-h infusion with bupivacaine paired with epinephrine in contrast to the unadulterated bupivacaine formulation. Additionally, the observed outcomes may vary depending on the concentration [28]. 

Particularly concerning drug carriers, none of the tested concentrations of lidocaine–prilocaine with nanostructured lipid–biopolymer hydrogels affected the cell viability of 3T3, HaCaT, and VERO cells [29]; however, a reduced toxic effect of lidocaine encapsulated in poly(epsilon-caprolactone) nanospheres compared to plain lidocaine was observed in 3T3 cells [8]. Bupivacaine-loaded alginate/chitosan and alginate/sodium bis(2-ethylhexyl) sulfosuccinate nanoparticles have shown similar protective effects [6]. The observed protective effects in these studies might be associated with the proportion of local anesthetic encapsulation, which ranged from 93.3% [8] to 75.6–85.7% [6]. These higher percentages of drug encapsulation compared to ours (51.8%) could lead to a lower concentration of free local anesthetic available to interact with cells. Discrepancies between these findings and those of the present study may be attributed to differences in the cell lines used.

The oral mucosa is recognized as an effective barrier, particularly when intact and undamaged [30]. This barrier primarily resides in the outer layers of the epithelium, a structure pivotal in limiting drug permeation [31]. The amorphous material with short-stacked lipid lamellae in the intercellular space, resulting from membrane-coating granules, contributes to the barrier’s characteristics [32]. Permeation studies focusing on oral applications often employ isolated buccal or esophageal epithelium as barriers. In our investigation, the pig esophageal epithelium was selected, given that pig tissues are frequently used due to their histologic structure, permeability, and lipid composition similarities [33,34].

Enhancers, particularly nanoparticles, have garnered significant attention for enhancing the buccal permeation of topical formulations [32]. In this study, we showcased the effectiveness of poly(epsilon-caprolactone) nanocapsules in augmenting lidocaine permeation across the pig esophagus epithelium. This enhancement aligns with previous studies, indicating a similar effect for an articaine formulation encapsulated within the same nanocarrier [10]. In addition, an absence of lag time was noted, indicating an instantaneous transport of the drug and potentially facilitating a rapid onset of pharmacological effects [35]. Similar results indicated that polymeric nanoparticles enhanced the flow rates of ibuprofen through pig skin in comparison to the drug in a buffered solution [36]. This enhancement in permeation was also noticeable when lidocaine and benzocaine were enclosed within liposomes based on phosphatidylcholine. Earlier studies emphasized a heightened flow, permeation pattern, and permeability coefficients for encapsulated local anesthetics in contrast to non-encapsulated commercial formulations (Xylocaina^®^ and Benzotop^®^, respectively) [37,38]. Further investigations revealed a significant and moderate association between in vitro flow and clinical effectiveness of topical anesthetics for both lidocaine and benzocaine, administered to either non-keratinized or keratinized oral mucosa [37,38]. This implies that in vitro permeation flow can serve as a useful indicator for anticipating the efficacy of topical anesthetics in the oral cavity.

Given the significant in vitro permeation flow of a local anesthetic, it is presumed that an efficient topical anesthetic effect on the oral mucosa may be achieved, regardless of the keratinization level of the epithelium. The observed high flow of lidocaine combined with polymeric nanocapsules presents promising prospects for future in vivo efficacy investigations in humans.

To evaluate the effectiveness of the anesthetic, the hind paw incision model in rats was selected. This model has been widely used to investigate pain and inflammation, as it is associated with postoperative pain in humans [39,40]. In this study, all animals displayed hypernociception, confirming the validity of this model. Within the initial assessment timeframe, which occurred 5 min after injection, no withdrawal response was observed in any of the animals, indicating paw anesthesia and a quick onset, as intended with the deliberately lower encapsulation achieved.

Ramos Campos et al. [8] observed an extended duration of analgesia ranging from 240 min to 420 min in a different anesthesia model evaluation involving paw pressure after a sciatic nerve blockade in mice. This was observed following the injections of lidocaine and lidocaine encapsulated in poly(epsilon-caprolactone) nanospheres. Variations in the duration of anesthesia noted in these studies may be attributed to their higher encapsulation efficiency, different nanocarrier evaluation, and the physiological tissue’s condition upon injection. In fact, nerve block techniques typically offer prolonged anesthesia compared to infiltration [2]. Moreover, inflammation diminishes anesthetic efficacy due to heightened tissue vascularity, lower tissue pH, and the peripheral sensitization of nerve fibers [2,40,41].

Despite the encapsulation of lidocaine in nanocapsules significantly increasing the duration of anesthesia in the present study, the combination with epinephrine demonstrated superior efficacy. We have chosen to use lidocaine with epinephrine to establish an effective positive control, as it is considered a gold standard in dental anesthesia for both the infiltrative and blockade techniques [4]. Its enhanced effectiveness is likely associated with the potent vasoconstrictive effects induced by epinephrine, which counteract and exceed the increased vascularity resulting from inflammation. Indeed, Khoshbaten and Ferrell [42] reported an intensified vasoconstrictive effect of epinephrine, potentially due to post-junctional adrenoceptor sensitization following inflammation induction in the rabbit knee. Moreover, Liu et al. [43] illustrated that the duration of anesthesia following a subcutaneous infiltration of 1% lidocaine could be doubled, even with a very low concentration of epinephrine, such as 1:3,200,000. In scenarios involving inflamed tissues, where achieving sufficient anesthesia is challenging, the encapsulation of lidocaine in nanocapsules did not surpass the efficacy of the combination with epinephrine. 

It is worth noting that the anesthesia model chosen in the present study may present a limitation, as we proposed a formulation for topical use. However, the literature lacks models to evaluate topical anesthetic efficacy. Therefore, if success is achieved in such a challenging model, the level of effectiveness in physiological conditions can be easily attained.

## 4. Materials and Methods

### 4.1. Rats

The Ethics Committee on Animal Experimentation of the Universidade Estadual de Campinas (UNICAMP) approved this experiment (#2636-1) and ensured compliance with EU Directive 2010/63/EU. Thirty-two male Wistar rats weighing 250 ± 50 g, obtained from CEMIB/UNICAMP, Brazil, accredited by ICLAS—International Council for Laboratory Animal Science, were used. They were housed in a controlled environment with a room temperature of 22 ± 1 °C and a 12 h light/dark cycle, and provided with free access to water and food.

### 4.2. Formulation of Lidocaine Encapsulated

Nanocapsules of poly(epsilon-caprolactone) with lidocaine were prepared by using the emulsification/diffusion (oil/water) method, as described previously [8]. Two solutions were prepared: (1) lidocaine base dissolved in 10 mL of acetone (Labsynth, Diadema, Brazil); and (2) 400 mg of poly(epsilon-caprolactone) (Sigma-Aldrich, St. Louis, MO, USA), 20 mL of chloroform (Sigma-Aldrich, St. Louis, MO, USA, and Labsynth, Diadema, Brazil, respectively), and 200 mg of Myritol 318 were mixed and sonicated at 100 W for 1 min [8]. Fifty milliliters of an aqueous solution containing 150 mg of polyvinyl alcohol (PVA, 86.09 g/mol—Sigma-Aldrich, St. Louis, MO, USA), was added to the pre-emulsion and sonicated for 8 min. The lidocaine-loaded poly(epsilon-caprolactone) nanocapsules, with an encapsulation efficiency of 83%, were obtained by evaporating the organic solvent. 

To achieve an encapsulation efficiency of 50%, an additional 298 mg of lidocaine was incorporated into the pre-existing formulation. Subsequently, the volume was adjusted to 16.6 mL to ensure uniformity between the amounts of lidocaine encapsulated within the nanoparticles and the unencapsulated portion. This procedural step of post-formulation lidocaine addition was imperative for facilitating an immediate action of the free anesthetic while enabling sustained release of the encapsulated variant over time, thereby maintaining prolonged anesthetic efficacy.

### 4.3. Lidocaine Analysis

Using high-performance liquid chromatography (HPLC) (Thermo Fisher Scientific Inc., Waltham, MA, USA), according to a previously validated method, lidocaine was measured [37]. A C18 reversed-phase column (5 μm, 150 × 4.60 mm, Gemini, Phenomenex) with a mobile phase comprising a mixture of acetonitrile (JT Baker, Phillipsburg, NJ, USA) and a buffer (25 mM NH_4_OH, adjusted to pH 7.0 with H_3_PO_4_) 60:40 (*v*:*v*) and a flow rate of 1.2 mL·min^−1^ was used. The injection volume was set to 20 µL and the detection wavelength was 220 nm at room temperature.

The specificity, linearity, accuracy, precision, detection limit (DL), and quantification limit (QL) were assessed [44]. The specificity of the analytical method was confirmed by injecting the lidocaine formulations, blank nanocapsules, and phosphate-buffered saline (PBS) (Sigma-Aldrich, St. Louis, MO, USA). Linearity was calculated with eight concentrations ranging from 0.1 to 200 µg/mL (y = 27807x + 9734.3; R^2^ = 0.9999).

The quantification (LQ = 1.04 μg/mL) and detection (LD = 0.31 μg/mL) limits were calculated based on the standard deviation of the response and slope. Precision and accuracy were determined intra-day and inter-day (in triplicate, on three consecutive days) using three concentrations (5, 50, and 200 µg/mL). The determined precision (RSD) was between 0.19 and 2.96%, and the level of accuracy was between 97.15 and 104.2% for the intra-day and inter-day evaluations, respectively [44].

### 4.4. Nanoparticle Characterization 

#### 4.4.1. Particle Size, Encapsulation Efficiency, and Stability

Before the analysis, the formulation was diluted (1:100, *v*/*v*) in deionized water (Milli-Q system, Millipore, Billerica, MA, USA), and the nanoparticle size distribution was determined, including the polydispersity index. To assess the physicochemical stability of lidocaine encapsulated in poly(epsilon-caprolactone) nanocapsules, we measured the pH of the formulation and nanocapsule diameter over 0, 30, 60, and 120 days. Throughout this duration, the formulation was stored in amber flasks at room temperature [18,45]. pH measurements were conducted using a calibrated potentiometer (Tecnal, Piracicaba, São Paulo, Brazil). The diameter of the nanocapsules was determined via dynamic light scattering using a ZetaSizer Nano ZS 90 analyzer (Malvern Instruments, Malvern, UK), operating at 25 °C and under a 90° angle. 

Encapsulation efficiency was assessed using previous studies [8,16,45]. In summary, the formulation was centrifuged with a regenerated cellulose filter (Microcon, Millipore, Billerica, MA, USA) (molecular exclusion pore size of 30 kDa). The encapsulation efficiency (%) was indirectly determined by subtracting the amount of lidocaine non-encapsulated from the total amount (100%) of lidocaine in the formulation, and plain lidocaine was evaluated by using HPLC.

#### 4.4.2. Differential Scanning Calorimetry (DSC)

DSC analyses were performed using a TA Q20 calorimeter equipped with a cooling system. Indium was employed for calibration, and samples were heated from −10 °C to 300 °C at a rate of 10 °C·min^−1^ under a nitrogen flow. Five milligrams of each sample were placed in aluminum pans for analysis, with an empty pan serving as a reference [46]. The samples subjected to analysis included poly(epsilon-caprolactone) nanocapsules (Nano), lidocaine encapsulated in poly(epsilon-caprolactone) nanocapsules (LDC-Nano), poly(epsilon-caprolactone) (PCL), a physical mixture of lidocaine and poly(epsilon-caprolactone) (PM LDC + PCL), and lidocaine (LDC).

### 4.5. MTT Assay

#### 4.5.1. Cell Culture

HaCaT cells were cultured similarly to a previous method employed by our group [9,10]. Dulbecco’s Modified Eagle Medium (DMEM) supplemented with 100 U/mL penicillin, 10% fetal bovine serum, and 100 μg/mL streptomycin (Vitrocell, Campinas, Brazil) were used. The cells were kept under a humidified atmosphere at 5% CO_2_/95% air at 37 °C. The medium was replaced every 2 days, and the culture was duplicated when cells achieved 70 to 80% confluence. The cells were then rinsed with phosphate-buffered saline (PBS) and treated with trypsin (0.25%) for 5 min. The trypsinization process was neutralized in DMEM supplemented with fetal bovine serum. Cells were centrifuged at 3000 rpm for 5 min (20 °C). The supernatant was removed. Fresh medium was added for the viability assay, and LDC, LDC-Nano, and LDC-Epi (prepared immediately before its use by adding 1:100,000 epinephrine—Sigma-Aldrich, St. Louis, MO, USA) to a lidocaine solution were tested. 

#### 4.5.2. Cell Viability

The MTT assay (Sigma-Aldrich, St. Louis, MO, USA) utilizes colorimetry to assess in vitro viability. This method relies on the reduction of tetrazolium (3-(4,5-Dimethylthiazol-2-yl)-2,5-diphenyltetrazolium bromide), MTT, into insoluble formazan by mitochondrial dehydrogenases, a process exclusive to living cells.

For this experiment, 5 × 10^4^ HaCaT cells/well were seeded into 96-well plates and incubated in 5% CO_2_ at 37 °C for 30 h. Subsequently, the cells were exposed to LDC-Nano, LDC-Epi, and LDC for 1 h. Lidocaine concentrations ranged from 0.08% to 1%. Control formulations (Nano, Epi, and DMEM) were also included. Cell viability was assessed after incubation with MTT (0.3 mg/mL) for 3 h at 37 °C. The resulting formazan crystals were dissolved in ethanol, and their absorbance was measured using a spectrophotometer (ASYS UVM 340 Biochrom LTDA, Cambridge, UK) at a wavelength of 570 nm under room temperature [42]. The results were expressed as the percentage of viable cells, and the IC_50_ was determined. This experiment was replicated eight times.

### 4.6. Permeation Assays

#### 4.6.1. Tissue Preparation and Selection

The esophagi were obtained post-mortem from pigs at a local slaughterhouse (Angelelli Fridge LTDA, Piracicaba, Brazil) and transferred in iced isotonic phosphate buffer (pH 7.4). Upon arrival, the esophagi were longitudinally incised and rinsed with saline. Subsequently, the mucosa was separated from the underlying muscular tissue and subjected to a water bath (60 °C for 60 s) to facilitate the detachment of the epithelium from the lamina propria [33].

The tissue was then positioned between the donor and receptor chambers of the Franz cells (Manual Transdermal System, Hanson Research Corporation, Chatsworth, CA, USA) with the connective side facing a cellulose filter (0.45 µm pore size, Millipore) to avoid any damage to the barrier and was allowed to equilibrate for 60 min. The chambers were filled with phosphate buffer to assess the integrity of the barrier by electrical resistance. It was measured using an alternating current of 100 mV (RMS) potency and 10 Hz frequency applied by a signal generator device (33210A, Agilent Technologies, Barueri, SP, Brazil) connected to Ag/AgCl electrodes [47]. The electrical current was measured at the receptor compartment using a multimeter. The tissue’s resistivity (*r*) was calculated in accordance with Ohm’s law (Equation (1)):

(1) $$r=\frac{\frac{P}{I}}{A}$$

where *P* = the potency of the alternated current (100 mV); *I* = intensity of the electrical current measured in µA; and *A* = the membrane area (1.77 cm^2^). Only tissues with an electrical resistance of higher than 3 KΩ/cm^2^ were considered adequate for the permeation test [47].

#### 4.6.2. In Vitro Permeation Assay

This experiment was conducted under constant magnetic stirring (400 rpm) for 5 h in Franz-type vertical diffusion cells with a permeation area of 1.77 cm^2^ and a receptor compartment volume of 7 mL. The selected epithelium was maintained between the donor and receptor chambers, which were filled with filtered/degassed phosphate buffer (pH 7.4). This experiment was carried out at 37 °C.

Samples of 300 µL were periodically withdrawn (0, 15, 30, 45, 60, 90, 120, 150, 180, 240, and 300 min) from the receptor chamber and instantaneously replaced with fresh buffer, which was considered for the dilution effect. These samples were analyzed by HPLC, as previously described.

The steady-state flux (J_ss_) and lag time (t_lag_) were obtained from the linear portion of the cumulative amount of lidocaine permeated per cm^2^ of esophagus epithelium (Q) versus time.

### 4.7. Anesthetic Effectiveness

The assessment of anesthetic efficacy utilized a rat model of surgical hypernociception development [48,49]. Initially, the animals were accommodated in cages comprising plastic dividers placed over a wired mesh floor platform for a 30 min acclimatization period. Beneath the platform, a mirror facilitated the observation of rat paws, allowing force application using a von Frey aesthesiometer (Insight Equipment Ltd., Ribeirão Preto, Brazil). Following this acclimatization, increasing forces ranging from 0.0073 to 0.456 N were applied every five minutes to the right hind paw to establish a baseline response. 

After the induction of anesthesia with isoflurane (Isoforine, Cristália Chemicals and Pharmaceuticals Ltd., Itapira, SP, Brazil), the animals underwent a 1 cm long × 3 mm deep incision on the plantar surface of the right paw, followed by three sutures using a 6-0 nylon (Brasuture Ind Imp Exp Ltd., São Sebastião da Grama, SP, Brazil).

The next day, the rats were positioned in the cage for a 30 min acclimatization, after which increasing forces were applied laterally to the wound. Animals showing at least a 20% reduction in the force required to elicit paw withdrawal were classified as hyperalgesic and underwent an assessment of anesthetic efficacy.

Laterally to the wound, these animals received an infiltration of 0.1 mL of 2% LDC-Epi, 2% LDC, and 2% LDC-Nano (*n* = 8 rats/group) or their respective controls NaCl (Ind. Equiplex farm., Brazil) or Nano. Five minutes post-infiltration, and every subsequent five minutes, the animals were submitted to force application (0.0073 to 0.456 N). The absence of a paw withdrawal reflex upon maximal force application indicated paw anesthesia [49].

### 4.8. Statistical Analysis

For our statistical analyses, the Shapiro–Wilk test was used for assessing normal distribution, and comparisons were performed by the Student’s *t*-test (diameter and polydispersity), unpaired *t*-test with Welch’s correction (in vitro permeation assay), non-linear fit analysis (MTT assay), log-rank Mantel–Cox test (anesthesia success), ANOVA, and Holm–Sidak’s multiple comparisons test (anesthesia duration). The significance level was set at 5% (α = 0.05). All statistical analyses were carried out using GraphPad Prism 6.0 software (GraphPad Software, Inc., La Jolla, CA, USA).

## 5. Conclusions

We introduced a suspension of lidocaine in poly(epsilon-caprolactone) nanocapsules, with lower encapsulation efficiency. The proposed formulation displayed favorable physicochemical features and showed comparable in vitro cytotoxicity to lidocaine alone. Additionally, this formulation exhibited enhanced anesthetic efficacy compared to lidocaine alone with a fast onset of action. The improved permeation parameters indicate their potential suitability for future evaluations as a topical formulation in the oral cavity.

## Figures and Tables

**Table 1:** pH, polydispersity index (PDI), mean diameter, and encapsulation efficiency (mean standard ± deviation) for only poly(epsilon-caprolactone) nanocapsules (Nano) and combined with lidocaine (LDC-Nano).

	pH	Polydispersity Index (PDI)	Mean Diameter (nm)	Encapsulation Efficiency (%)
LDC-Nano	8.1 ± 0.21 ***	0.08 ± 0.01 **	557.8 ± 22.7 *	51.8 ± 1.87
Nano	6.3 ± 0.21	0.16 ± 0.02	530.5 ± 9	-

* *p* = 0.13; ** *p* = 0.008; and *** *p* < 0.0001. *t* test.

**Table 2:** Mean values (± SD) of the steady-state flux (J_ss_), enhancement ratio (ER), cumulative amount of lidocaine permeated per cm^2^ of esophageal epithelium after 300 min (Q_5h_), and correlation coefficient (r) of the linear portion of the curve for both formulations (LDC and LDC-Nano) are provided.

Formulation	J_ss_ (μg·cm^−2^·h^−1^)	ER *	Q_5h_ (μg·cm^−2^)	ER ^#^	r
LDC	41.53 ± 9.04	1.0	224.80 ± 40.46	1.0	0.98 ± 0.003
LDC-Nano	309.71 ± 55.41 ***	7.46	1485.76 ± 394.11 **	6.61	0.993 ± 0.003

*** *p* < 0.0001; ** *p* = 0.0002; Unpaired *t*-test with Welch’s correction. Each parameter was analyzed separately. * Enhancement ratio between the steady-state flux of LDC in comparison to LDC-Nano. ^#^ Enhancement ratio between the cumulative amount of lidocaine permeated per cm^2^ of esophageal epithelium after 5 h of LDC in comparison to LDC-Nano.

## Data Availability

The data presented in this study are available from the authors on request.
